# Worldwide productivity and research trend of publications concerning intestinal polyps: A bibliometric study

**DOI:** 10.1097/MD.0000000000036507

**Published:** 2024-01-12

**Authors:** Sha Si, Letian Shou, Qi Gao, Wenyan Qin, Dan Zhao

**Affiliations:** a Department of Food Science and Engineering, Ningbo University, Ningbo, China; b School of Marine Science, Ningbo University, Ningbo, China; c Yinzhou No. 2 People’s Hospital, Ningbo, China.

**Keywords:** bibliometric analysis, CiteSpace, hot topics, intestinal polyps, visualization

## Abstract

There is a significant relationship between intestinal polyps and colorectal cancer, and in recent years, research on intestinal polyps has been rapidly developing around the world. However, there is still a lack of adequate quantification and analysis of publications in this field. The aim of this study was to perform a comprehensive bibliometric analysis of publications related to intestinal polyps over the past 20 years. To enhance the understanding of current research hotspots and potential trends, and to point out the direction of future research. Publications related to intestinal polyps were retrieved from the Science Citation Index Expanded in Web of Science Core Collection. the Bibliometric online analysis platform (https://bibliometric.com/app), the Bibliometrix Package, and the CiteSpace are used for bibliometric analysis and visualization, including the overall range of annual output and annual citations, country-region analysis, author and institution analysis, core journal analysis, reference and keyword analysis. Prior to 2017, the amount of research on intestinal polyps was slow to grow, but it picked up speed after that year. In 1019 journals, 4280 papers on intestinal polyps were published in English. The journal with the highest productivity was Gastrointestinal Endoscopy (189, 4.42%). United States (1124, 26.26%), which is also the hub of collaboration in this subject, was the most productive nation. Mayo Clinic (n = 70, 1.64%) is the most productive institution. Intestinal microbiota, endoscopic mucosal resection, gut microbiota, deep learning, tea polyphenol, insulin resistance and artificial intelligence were current hot subjects in the field. Studies of intestinal polyps increased significantly after 2017. The United States contributed the largest number of publications. Countries and institutions were actively cooperating with one another. artificial intelligence is currently an emerging topic.

## 1. Introduction

Colorectal polyps are bulging lesions of colorectal mucosa protruding into the lumen, which can be divided into inflammatory polyps, adenomatous polyps, dysplastic polyps, and hyperplastic polyps. Adenomatous polyps will develop into colorectal cancer through adenomatous cancer sequence, and it is the main component of the malignant polyp.^[1]^ The incidence of intestinal polyps is related to gender,^[2]^ smoking history,^[3]^ drinking history,^[4]^ obesity,^[5]^ *Helicobacter pylori* infection,^[6]^ and related family history.^[7]^ Colorectal cancer is the second deadliest cancer and the third most common cancer in the world.^[8]^ As the sequential evolution of polyp-adenoma-cancer theory becomes a consensus, early detection of precancerous polyps and early removal of precancerous lesions are crucial to reduce the incidence and mortality of colorectal cancer. Colorectal cancer has no obvious discomfort symptoms in the early stage, and it is mostly in the middle and late stage when it is found. Therefore, early detection of colorectal polyps is a key part of rectal cancer prevention. With the change in population composition and the progress of medical technology, the diagnosis and treatment pattern and epidemiological characteristics of colorectal polyps may change accordingly. Therefore, it is particularly important to describe and forecast the research status, progress and development trend of colorectal polyps in the world.

Bibliometric is an interdisciplinary science that quantitatively analyzes all knowledge carriers by mathematical and statistical methods.^[9]^ Through the measurement of literature in a particular field, bibliometrics research aims to uncover important pathways and knowledge turning points in the evolution of a discipline. It also analyzes potential dynamic mechanisms of discipline evolution and identifies the frontiers of discipline development by creating a series of visual maps.^[10]^ Bibliometric can make qualitative and quantitative analysis of the country, journal, publication year, keywords and author of publications, and provide the main research fields, development trends or emerging research directions of a particular subject, which can help scholars discover the development characteristics of this field, and indicate the future research direction.^[11]^ Bibliometric has been widely used as an auxiliary research method in many disciplines.^[12]^ To the best of our knowledge, no reports on colorectal polyps from a bibliometric perspective have been published to yet, despite the continual increase in the number of publications on the condition. In this study, bibliometric and visualization analyses were performed using CiteSpace 6.1.R2 and Bibliometrix 4.1.0 packages, an online bibliometric analysis platform, along with the data source of colorectal polyp-related publications from the Web of Science Core Collection database for the past 20 years. By mapping scientific knowledge, the current research status, hotspots and trends in this research area were visualized to provide valuable references for further research.

## 2. Materials and methods

### 2.1. Data source and search strategy

We conducted a literature search of intestinal polyps over the past 20 years (January 2003 to December 2022) on the Science Citation Index Expanded in Web of Science Core Collection database. The retrieval formula is as follows: TI = intestinal or TI = colon* or TI = colorectal or TI = rectum or TI = gut and TI = polyp*. The symbol * was used to represent zero or more characters. The language setting of the article is English only. The document types included in the present study are article and review article. All the above operations were completed on January 10, 2023 to avoid errors due to data updates. According to the requirements of bibliometrics tools used, full records and cited references in the corresponding format are derived, including the number of papers and citations, title, author, year of publication, country, institution, journal, keyword and reference, for bibliometric analysis. Ethical approval was not necessary because the data did not include any private information about patients.

### 2.2. Statistical analysis

In this study, the Bibliometric Online Analysis Platform (https://bibliometric.com/app), the Bibliometrix 4.1.0 Package based on the R language, and the CiteSpace (version 6.1.R2) were used for bibliometric analysis. The publications were downloaded as “download_xxx.txt” and exported as a plain text file with “full record and cited references” from the Web of Science Core Collection database, and then imported the downloaded files into the CiteSpace 6.1.R2 software. Meanwhile, all documents were exported using the “full record and cited references” format in UTF-8 and then imported into the Bibliometrics Online Analysis Platform and Bibliometrix package respectively. The Bibliometrics Online Analysis Platform was used to analyze cooperation networks between countries. The Bibliometrix Package is a feature Package of R language for bibliometric analysis.^[13]^ We used the Bibliometrix package to analyze changes in annual publications and average annual citations, publications and citations in the top 10 countries and regions, and annual publications and citations analysis of top authors. CiteSpace was used for visual analysis of emerging trends and dynamics of scientific research in specific research areas.^[14]^ We used it to analyze institutional collaboration networks, journal citation network graphs, reference bursts, and keyword bursts. The CiteSpace software was set with the following parameters: Time span: January 2003 to December 2022; Years per slice: 1; node types: institution, cited journal, reference, keyword; pruning: pathfinder and pruning sliced networks. The rest of the settings remained the software defaults.

## 3. Results

### 3.1. General information

**Figure 1.**
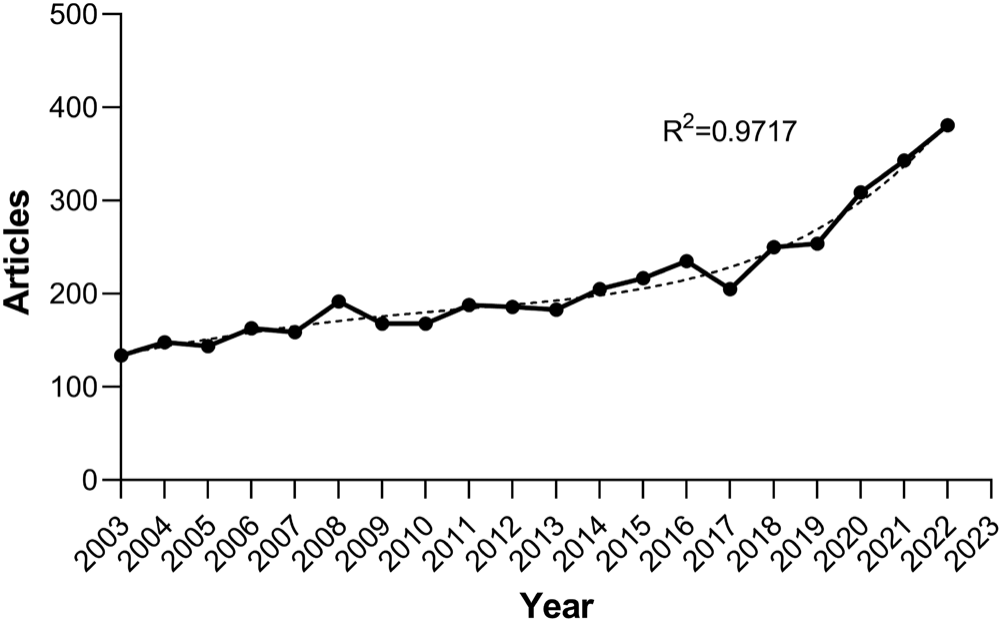
Annual scientific production in the field of intestinal polyps research as of December 31, 2022.

A total of 4280 publications on intestinal polyps were retrieved from Web of Science Core Collection, including 3858 articles and 422 review articles. Figure 1 shows the annual scientific production of intestinal polyps from January 1, 2003 to December 31, 2022. Figure 1 shows that annual scientific publications on intestinal polyps have shown a generally upward trend over the past 2 decades. Publication growth was relatively slow until 2017 and increased significantly after 2017. The number of publications that have been published since 2017 has increased quickly, suggesting that academics have been paying more and more attention to colorectal polyps and that the study of these lesions has grown in popularity recently. The average annual production of publications was around 200, with the lowest number of publications in 2003 (n = 134, 3.13%), and from 2014 onwards, the number of annual publications exceeded 200 (n = 205, 4.79%), with the maximum number of annual publications in 2022 (n = 381, 8.90%). The association between the volume of publications and the year was assessed using a four-term polynomial model. With a high coefficient of determination (R^2^ = 0.9717), it was demonstrated that the fitted polynomial curve closely matched the annual literature growth trend. The development of colorectal polyps screening methods and the advancement of research are the causes of this increase. Annual articles will likely keep expanding in the upcoming years, according to the fitting curve.

**Figure 2.**
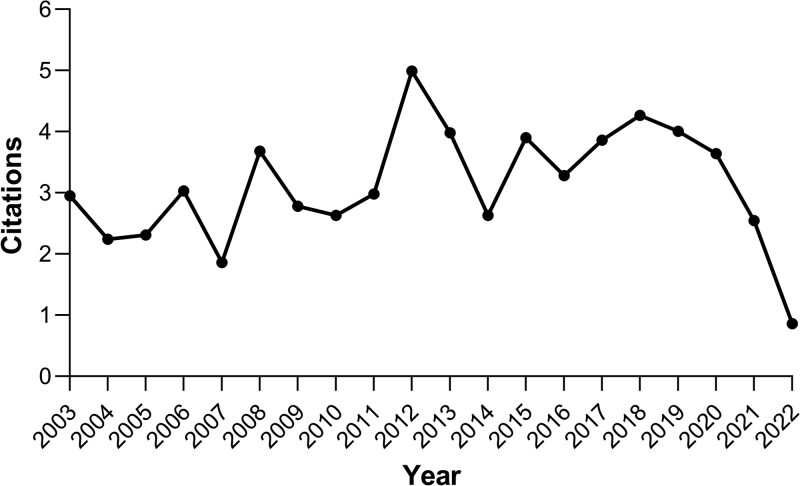
Average article citations per year in the field of intestinal polyps research as of December 31, 2022.

The citations of these 4280 publications were analyzed as a whole, and the publications were cited 130,016 times, an average of 30.38 times per article. Figure 2 shows the average annual article citations. From Figure 2, we can see that the overall annual article citation rate is relatively stable, with more local citations. A peak in citations was reached in 2012, after which the average annual citation fluctuated more gently.

### 3.2. Country region analysis

**Figure 3.**
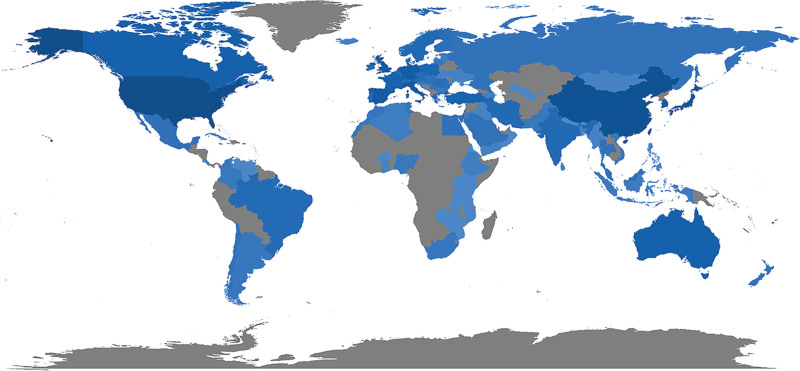
Geographical distribution of global output in intestinal polyps research field.

From 2003 to 2022, publications on intestinal polyps were published in 89 countries. The global publication productivity is shown in Figure 3. The global map shows blue countries and regions where there are corresponding authors. The intensity of the blue color on the map corresponds to the number of publications in the country or region; the darker the blue color, the more publications have been published there. The United States, China, and Japan are clearly the 3 nations with the darkest color. In fact, these 3 countries account for the biggest percentage of publications (26.26%, 14.74%, and 9.42%, respectively). The countries with a high number of publications (top 5) of corresponding authors are, in order, the United States, China, Japan, the United Kingdom, and Italy, as shown in Table 1. The total number of publications from these 5 countries accounted for 78.37% of the top 10 countries. This situation might be associated with socioeconomic factors of the country, such as gross domestic product, population, etc, or with the country’s relevant interests in the field of intestinal polyps research. Table 1 also shows the top 10 most cited countries or regions. The top 5 countries in terms of total citations were the United States, Japan, China, the United Kingdom, and Spain. The total citations for these countries demonstrates their relative breadth in the field. The country with the most total citations was the United States, with 47,951 citations. These 5 countries accounted for 78.02% of the total citations of the top 10 countries. In addition, New Zealand publications had the highest average number of citations, with an average of 84.83 citations, followed by Belgium, The United Arab Emirates, Austria, and Spain. The average citation volume of these 5 countries accounted for 51.1% of the average citation volume of the top 10 countries, as shown in Table 2. Despite having less publications overall, Netherlands, and Belgium had higher average citation counts than the majority of other nations, a sign of the high caliber of the research done in these nations. As the top 5 countries with the number of publications, the average article citation of America, China, Japan, the United Kingdom, and Italy are not too high.

**Table 1:** Top 10 corresponding author’s countries and total citations countries in intestinal polyps research field.

Country	Articles (%)	Total citations
USA	1124 (26.26%)	47,951
CHINA	631 (14.74%)	10,223
JAPAN	403 (9.42%)	10,501
UNITED KINGDOM	243 (5.68%)	9408
ITALY	223 (5.21%)	6917
KOREA	190 (4.44%)	2779
GERMANY	171 (4.00%)	5237
SPAIN	149 (3.48%)	7109
NETHERLANDS	130 (3.04%)	6147
CANADA	83 (1.94%)	2915

**Table 2:** Average article citations of the major participating countries or regions.

Country	Average article citations
NEW ZEALAND	84.83
BELGIUM	68.08
U ARAB EMIRATES	60.00
AUSTRIA	54.60
SPAIN	47.71
NETHERLANDS	47.28
USA	42.66
UNITED KINGDOM	38.72
NORWAY	36.83
COLOMBIA	36.33

**Figure 4.**
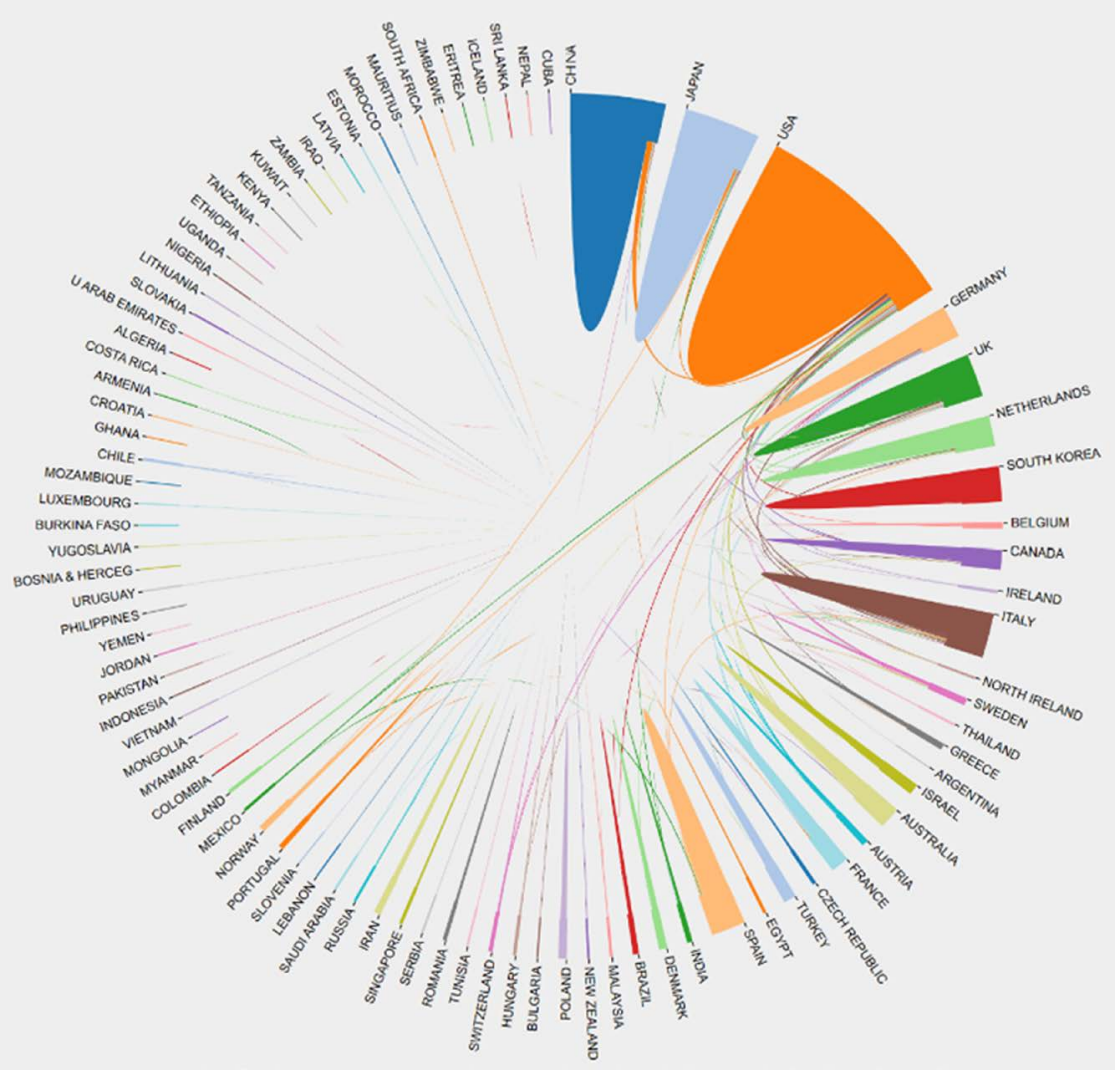
Countries collaboration map in intestinal polyps research field.

Figure 4 shows the countries collaboration map in the field of intestinal polyps. With distinct color blocks representing different countries and regions and the size of the color blocks showing the number of published articles, it demonstrates the cooperative position among various countries and regions. The degree of cooperation is represented by the lines joining the color blocks, and the strength of the relationship is indicated by the thickness of the lines.^[15]^ There are great differences in cooperation between different countries. As shown in Figure 4, the United States is the central region for collaboration in this field, and it has collaborative relationships with more than 30 other countries. In addition, Japan, the United Kingdom, China, Italy, Germany, and Spain also work closely with other countries. And some countries, such as Kenya and Zambia, Eritrea hardly cooperate with other countries. The figure illustrates that China is a high-yielding nation that collaborates more with the United States but less with other nations/regions. It should increase its exchanges and collaboration with other nations/regions.

### 3.3. Author and institutional analysis

**Figure 5.**
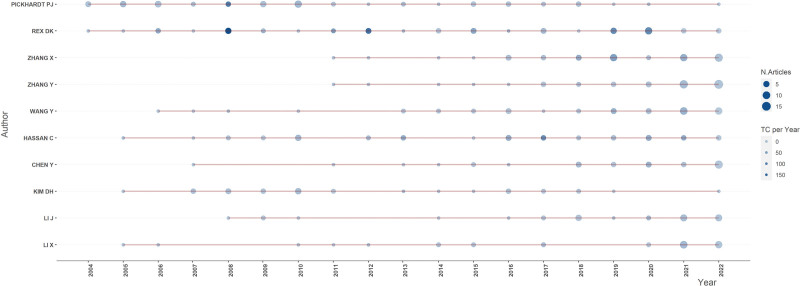
Top 10 most relevant authors’ production in intestinal polyps research field.

In our analysis of 4280 publications, there were a total of 18,384 related authors. Figure 5 shows the results for the top 10 authors with the most publications. Of these, the most relevant author is Douglas K. Rex of Indiana University, who has published 53 articles in the field of intestinal polyps. The red horizontal line in Figure 5 is the timeline of the author’s publications, and the size of the circle is proportional to the number of publications, which means that the larger the circle, the greater the number of publications related to the author in that year. The color of the circle indicates the total citations of the publications, and the darker the color, the higher the citations of the author’s publications in that year. Figure 5 shows that over the past decade, an increasing number of researchers have dedicated their research to the field of intestinal polyps.

**Figure 6.**
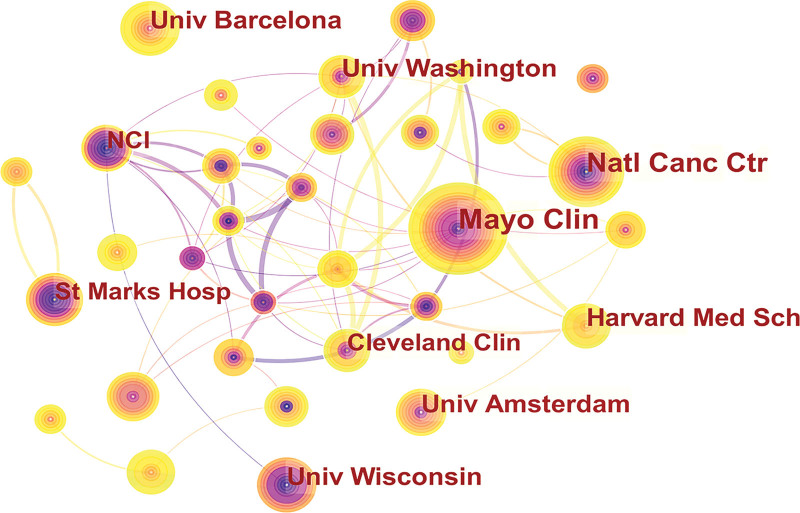
The network map of institutes for intestinal polyps research.

A total of 4804 institutions published publications on intestinal polyps. The top 10 institutions with the highest output in intestinal polyps research are shown in Table 3. Mayo Clinic in the United States (n = 70, 1.64%) was the leading institution in terms of output, followed by the Natl Canc Ctr in Japan (n = 60, 1.40%), and the University of Wisconsin in the United States (n = 45, 1.05%). Figure 6 shows the collaborative relationships between institutions with at least 20 publications. As can be seen from Figure 6, Mayo Clinic is at the absolute center and has a closer partnership with other institutions. Following closely behind are Natl Canc Ctr and the University of Washington, who also have some collaboration with other institutions.

**Table 3:** Top 10 sources of publications in the field of intestinal polyps research.

Affiliation	Articles (%)
MAYO CLIN	70 (1.64%)
NATL CANC CTR	60 (1.40%)
UNIV WISCONSIN	45 (1.05%)
HARVARD MED SCH	43 (1.00%)
UNIV BARCELONA	41 (0.96%)
UNIV AMSTERDAM	40 (0.93%)
UNIV WASHINGTON	39 (0.91%)
NCI	39 (0.91%)
ST MARKS HOSP	35 (0.82%)
CLEVELAND CLIN	34 (0.79%)

### 3.4. Sources analysis

**Figure 7.**
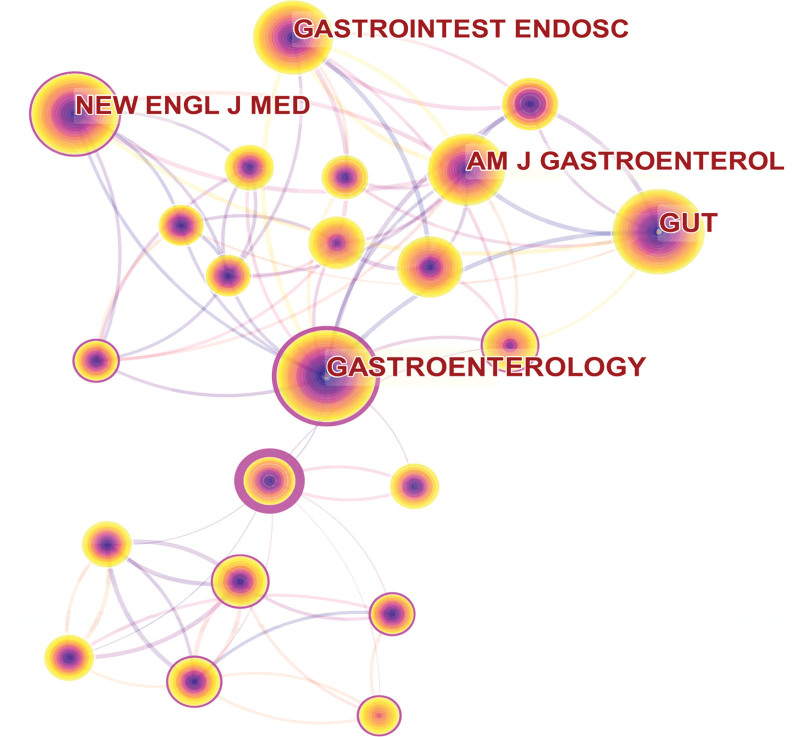
The network map of journals in the field of intestinal polyps.

All publications on intestinal polyps were published in 1019 journals. Table 4 shows the top 10 sources with the highest productivity in the field of intestinal polyps research. Publications published by these journals accounted for 18.34% of the total number of publications. Gastrointestinal endoscopy is the most widely published journal in the field of intestinal polyps with 189 articles (4.42%) and the impact factor (IF) of this journal is 10.396. The second source journal is the World Journal of Gastroenterology, which published 88 articles (IF = 5.374, 2.06%). Other top journals include Endoscopy with 86 articles (IF = 9.776, 2.01%), International Journal of Colorectal Disease with 65 articles (IF = 2.796, 1.52%), and Diseases of the Colon & Rectum with 63 articles (IF = 4.412, 1.47%). The network of journals with at least 500 citation frequencies is depicted in Figure 7, where it is clear that GASTROENTEROLOGY (IF = 33.883) is a highly significant publication that is frequently cited by other journals. Additionally, GUT (IF = 31.793), GASTROINTEST ENDOSC (IF = 10.396), ENDOSCOPY (IF = 9.776), and AMERICAN JOURNAL OF GASTROENTEROLOGY (IF = 12.045) also have co-citation connections with many other publications and have a substantial impact in this field. For researchers in the field of intestinal polyps, these top journals are especially significant when they submit their manuscript.

**Table 4:** Top 10 journals in intestinal polyps research.

Sources	Articles (%)	Impact factor
GASTROINTESTINAL ENDOSCOPY	189 (4.42%)	10.396
WORLD JOURNAL OF GASTROENTEROLOGY	88 (2.06%)	5.374
ENDOSCOPY	86 (2.01%)	9.776
INTERNATIONAL JOURNAL OF COLORECTAL DISEASE	65 (1.52%)	2.796
DISEASES OF THE COLON \& RECTUM	63 (1.47%)	4.412
JOURNAL OF AGRICULTURAL AND FOOD CHEMISTRY	62 (1.45%)	5.895
GASTROENTEROLOGY	60 (1.40%)	33.883
CLINICAL GASTROENTEROLOGY AND HEPATOLOGY	59 (1.38%)	13.576
DIGESTIVE DISEASES AND SCIENCES	58 (1.36%)	3.487
SURGICAL ENDOSCOPY AND OTHER INTERVENTIONAL TECHNIQUES	55 (1.29%)	3.453

### 3.5. Most local cited references and references with the strongest citation bursts

**Figure 8.**
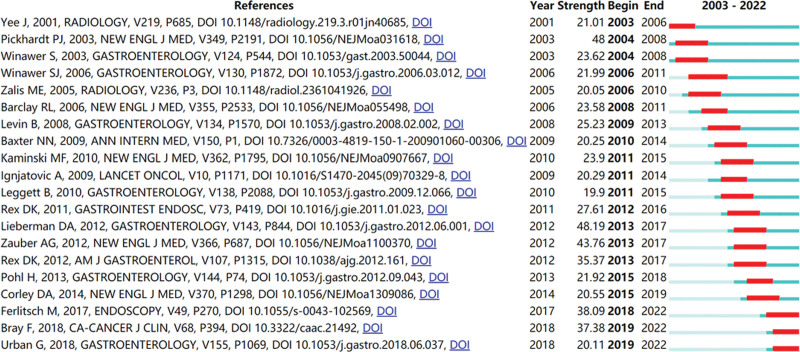
Top 20 references with strongest citation bursts.

References with citation bursts are those that are repeatedly cited throughout time, according to the definition.^[16]^ Table 5 shows the top 10 most local cited references associated with studies of intestinal polyps, with 3 reviews and 7 articles among these 10 references. Of these, the reference by Sidney and Ann had the most local citations of these,^[17]^ followed by the references by Ann et al,^[18]^ David et al,^[19]^ Perry et al,^[20]^ Douglas et al,^[21]^ and the other 5 references^[22–26]^ had local citations ranging from 125 to 154. Figure 8 shows the top 20 references with the highest number of citation bursts (defined as lasting at least 3 years). The red line shows the duration from the start of the co-cited reference to its conclusion, and the blue line shows the year of the outbreak. “Strength” is the equivalent of burst strength. Higher values correspond to higher strength and more influence of the publication.^[27]^ Half of these references started having citation bursts between 2010 and 2015, with the earliest reference^[28]^ with a citation burst appearing in 2003.

**Table 5:** Top 10 most local cited references related to intestinal polyps research.

Cited references	Citations	Type of reference
WINAWER SJ, 1993, NEW ENGL J MED, V329, P1977^[17]^	439	Article
ZAUBER AG, 2012, NEW ENGL J MED, V366, P687^[18]^	296	Article
LIEBERMAN DA, 2012, GASTROENTEROLOGY, V143, P844^[19]^	229	Article
PICKHARDT PJ, 2003, NEW ENGL J MED, V349, P2191^[20]^	201	Article
REX DK, 1997, GASTROENTEROLOGY, V112, P24^[21]^	157	Article
VOGELSTEIN B, 1988, NEW ENGL J MED, V319, P525^[22]^	154	Article
FEARON ER, 1990, CELL, V61, P759^[23]^	140	Review
REX DK, 2012, AM J GASTROENTEROL, V107, P1315^[24]^	139	Review
VAN RIJN JC, 2006, AM J GASTROENTEROL, V101, P343^[25]^	132	Review
REX DK, 2011, GASTROINTEST ENDOSC, V73, P419^[26]^	125	Article

### 3.6. Keywords analysis

Analyzing the co-occurrence of keywords in a paper can be beneficial in identifying research hotspots within the subject, as they are the substance of the topic and content.^[29]^ The most frequent author’s keywords and their frequency of occurrence in intestinal polyps publications are shown in Table 6. Three keywords with the highest frequency are colorectal cancer, colonoscopy, and polyphenols, which represent a major research scope in the field of intestinal polyps. Among them, the keywords related to the treatment of intestinal polyps were colonoscopy and polypectomy.

**Table 6:** Top 10 most frequent author’s keywords in intestinal polyps research.

Words	Occurrences
Colorectal cancer	450
Colonoscopy	428
Polyphenols	207
Polyp	171
Colon	169
Colon cancer	148
Polypectomy	138
Polyps	127
Adenoma	124
Gut microbiota	122

**Figure 9.**
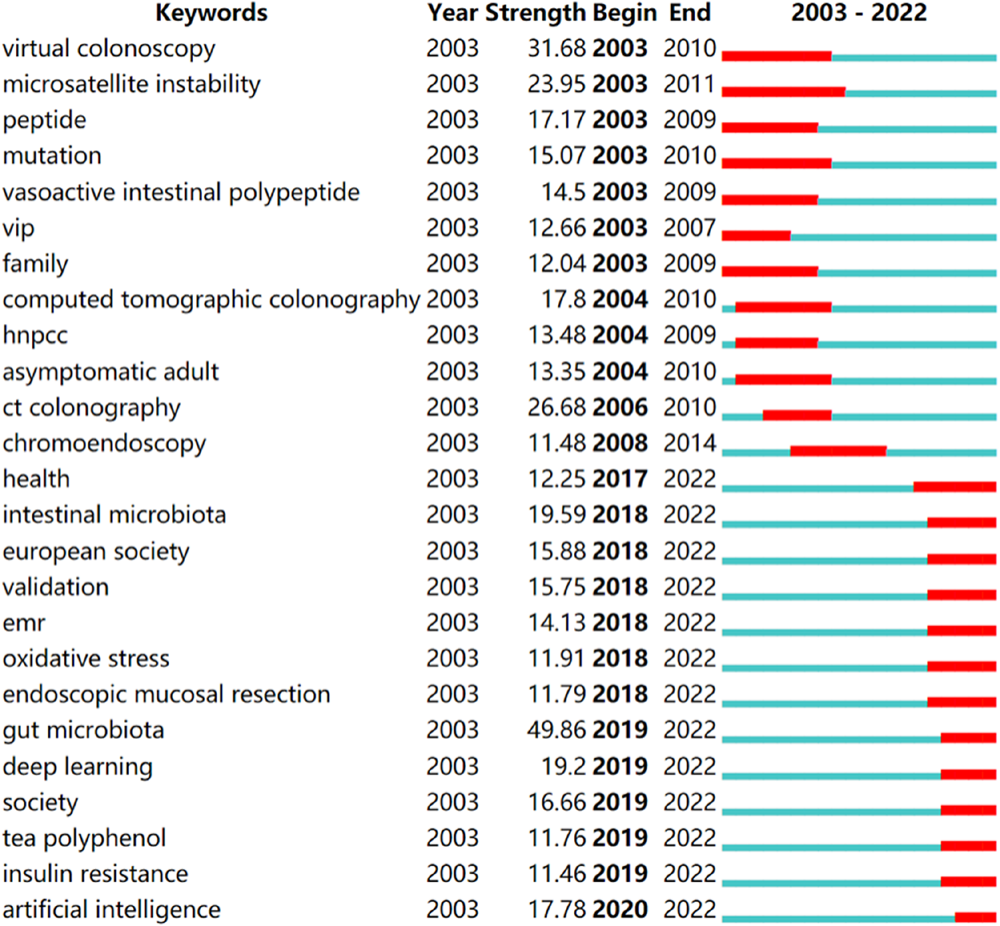
Top 25 keywords with strongest citation bursts.

The burst of keywords can detect the current research frontiers and hotspots in the research field. Figure 9 shows the top 25 keywords with the strongest citation bursts in the field of intestinal polyps research. In Figure 9, the blue line represents the time line. The burst period is the red line segment on the blue timeline, which represents the start and end time of the research frontier, that is, the period when the occurrence and frequency of a certain keyword increased rapidly.^[30]^ We found that the “gut microbiota” had the highest burst intensity in the last decade. Current research trends mainly include intestinal microbiota, endoscopic mucosal resection, gut microbiota, deep learning, tea polyphenol, insulin resistance, and artificial intelligence. Among them, artificial intelligence is the hotspots newly appeared in the past 3 years.

## 4. Discussion

The bibliometric study of intestinal polyps is conducive to summarize the research trends in this field in recent years. We conducted a literature search in Science Citation Index Expanded on intestinal polyps in the last 20 years and finally retrieved a total of 4280 relevant publications. Our findings show a steady overall increase in the number of publications related to intestinal polyps between 2003 and 2022, indicating the growing interest of researchers in the field of intestinal polyps. Prior to 2017, the number of annual scientific publications was in the range of 150, with a slow growth rate. Starting in 2017, the number of annual scientific publications increased rapidly, particularly during the period 2019 to 2022. The probable reasons for the sharp increase might be the great improvement in the diagnosis and treatment methods of intestinal polyps; the increased national attention and financial support in the field of intestinal polyps; and the increasing number of people who are aware of the knowledge related to intestinal polyps and devoted to the related research. And as can be seen, the average article citations reached its first peak of 5 in 2012 and remained consistently high after 2014. Overall, these findings indicate that the field of intestinal polyps has achieved substantial advancements in the past ten years, particularly in 2012. The findings show that the field of intestinal polyps research has drawn a growing number of researchers and garnered a great deal of attention, leading to a prolific output of research accomplishments and encouraging the area’s rapid development.

We analyzed a geographical distribution of publications in the field of intestinal polyps. Although as high as 89 countries have contributed to research in the field of intestinal polyps during the last 20 years, only a few of them have produced the majority of publications. Over half of the publications were published by 5 countries (USA, China, Japan, UK, and Italy), which reveals the important role played by these countries in the research in this field. The high total citation rating of the Netherlands and Belgium reflects the excellent caliber of the research conducted in these countries. In general, developed countries collaborated more with other countries, among which the United States collaborated most closely with other countries and China also cooperated more with other countries. There were 446 publications contributed by the top 10 institutes, representing 10.42% of the publications included in the study. Among the top 10 institutions, 7 were from the USA. The USA led in the number of institutions in this area of research. The top 10 institutions do not include institutes from countries with a high number of publications, such as Italy. Collaboration among institutes is relatively frequent. Even though China is a very productive nation, the lack of top-ranked research institutions in the nation suggests that Chinese research institutions lack core competitiveness in the global arena. To increase their influence, Chinese research institutions need to enhance their own research capabilities and forge close partnerships with other international research institutions. In addition to promoting more major research advances and breakthroughs via cooperation, close interactions and cooperation among nations and research institutions can direct research to stay pace with international research frontiers and hotspots.

There were 18,384 authors associated with the 4280 publications, with the most relevant author being Douglas K. Rex of Indiana University, who published 53 articles on the field of intestinal polyps between 2004 and 2022. 4280 publications were published in 1019 journals, but only 14.72% of journals published more than 5 publications and as many as 70.56% of journals published 1 or 2 publications, indicating that although a large number of journals have contributed to the publication of intestinal polyps research, only a few journals persisted in publishing research related to intestinal polyps. Seven of the top 10 journals were from the United States, and 6 of them had impact factors higher than 5.000, indicating that journals from the United States publish research in the field of intestinal polyps more frequently and that these journals also had high impact factors. Three of the top 5 co-cited journals are also among the top 10 most productive journals, and 80% of them have impact factors above 10.000, indicating that publications published in high-impact journals are cited more frequently. This phenomenon may be caused by the high standards for topic selection and published article quality in high-impact journals. These publications also have higher social media distribution rates and more attention-getting potential. Consequently, if a researcher wants to publish research in this field, he should first choose high-productivity journals for submission and refer to the papers published in highly cited journals.

Publications with citation bursts can help to characterize the evolution of a field.^[31]^ A study by Joanna^[28]^ from 2001 was the first to identify a citation burst; it occurred between 2003 and 2006. This study discovered that computed tomographic colonography is highly sensitive for identifying colorectal polyps that are clinically significant. An article written by Michal et al that was published in 2010 had the strongest of the citation bursts that started in 2011.^[32]^ This study showed that, following screening colonoscopy, the rate of adenoma discovery was an independent predictor of risk of septal colorectal cancer. The strongest burst in 2013 was caused by a 2012 report by David^[19]^ on guidelines for screening and post-polypectomy colonoscopy surveillance. The authors stressed the significance of maintaining close watch on important quality indicators as part of a colonoscopy screening and surveillance program. The citation explosion from 2015 and also the highest burst for that year was a 2013 article by Heiko et al^[33]^ about the incidence of incompletely resected tumor polyps. This study suggested that incomplete removal of colon polyps may lead to the development of colon cancer after colonoscopy, and efforts should be made to ensure complete removal. The strongest burst in the last 5 years was an article published in 2017. This guideline addressed the main questions regarding the practical aspects of polypectomy and endoscopic mucosal resection, providing information and a basis for this essential technique in colonoscopy and colorectal cancer prevention.^[34]^ In addition, the emergence of keywords can also describe the research frontiers and hot spots in the field. Intestinal microbiota, endoscopic mucosal resection, gut microbiota, deep learning, tea polyphenol, insulin resistance and artificial intelligence are the current research frontiers. The recent 5 years have seen “artificial intelligence” emerge as new hot issues. We need to continue to focus on the diagnosis and removal of colorectal polyps, which is also a hotspot of current research. Colorectal cancer can be avoided by promptly identifying and removing colorectal polyps. The primary application of colonoscopy is in the differential diagnosis of polyps. AI technology is expected to enhance the performance of colonoscopists, increase diagnostic precision, and enhance the ability to identify, classify, and isolate polyps during colonoscopy procedures. These changes may lead to higher rates of adenoma identification and, eventually, lower rates of colorectal cancer morbidity and death.

This article uses 3 visualizations to identify current hot areas, authors, countries, and institutions publishing and cooperating in the subject of intestinal polyps in order to better understand the state of research on these lesions. However, this study still has several flaws. Firstly, Web of Science Core Collection database alone was searched for data rather than various other databases like Embase or Scopus. But one needs to note that Web of Science Core Collection database is the most frequently used technique for scientometric analyses.^[16]^ Additionally, evaluating data from several databases at once presents significant challenges for current scientometric tools. Secondly, all data were gathered by scientometric tools rather than artificially extracted by the authors in systematic review overviews or meta-analyses.^[35,36]^ Therefore, bias in our findings may also present. For instance, the possibility of homonyms for authors, as well as several names for the same structures, various units for the same author, etc cannot be completely ruled out, yet these data cannot be accurately acquired by the current tools, and potential inaccuracies still exist. The advancement of machine learning, data analytics, and natural language processing may in the future provide solutions to these issues.^[37]^ Furthermore, due to all of the listed publications are in English, there may be bias in the selection. Therefore, investigations of intestinal polyps published in other languages may not be applicable to these findings. Lastly, it’s crucial to understand that scientific research initiatives in this area don’t always correspond to their practical application and influence in this area. The aforementioned conclusions just reflect the most recent developments in academic thought, which is the major focus of this paper.

## 5. Conclusions

Research on intestinal polyps has increased significantly over the past 20 years, especially between 2017 and 2022. Globally, the country with the highest research output in this field was the United States, which was also the center of collaboration in this field. There was an active collaboration among several developed countries. One of the most relevant authors was Douglas K. Rex of Indiana University, who published 53 articles in the field of intestinal polyps. The Vanderbilt University, University of Wisconsin, and the University of Amsterdam were the 3 most productive institutes. Intestinal microbiota, endoscopic mucosal resection, gut microbiota, deep learning, tea polyphenol, insulin resistance and artificial intelligence were probably the hottest topics at the moment.

## Author contributions

**Conceptualization:** Wenyan Qin, Dan Zhao.

**Formal analysis:** Sha Si.

**Investigation:** Sha Si.

**Resources:** Sha Si.

**Software:** Letian Shou.

**Visualization:** Qi Gao.

**Writing – original draft:** Sha Si.

**Writing – review & editing:** Sha Si, Wenyan Qin, Dan Zhao.
